# The impact of the COVID-19 pandemic on the antibiotic consumption and resistance in Montenegro

**DOI:** 10.1093/eurpub/ckaf167

**Published:** 2025-09-13

**Authors:** Gordana Mijovic, Maja Raicevic, Milena Lopicic, Slavica Markovic, Marina Jaksic

**Affiliations:** Faculty of Medicine, University of Montenegro, Podgorica, Montenegro; Faculty of Medicine, University of Montenegro, Podgorica, Montenegro; Institute for Children’s Diseases, Clinical Centre of Montenegro, Podgorica, Montenegro; Institute for Public Health of Montenegro, Podgorica, Montenegro; Statpro DOO, Podgorica, Montenegro; Faculty of Medicine, University of Montenegro, Podgorica, Montenegro; Institute for Children’s Diseases, Clinical Centre of Montenegro, Podgorica, Montenegro; Center for Science, Clinical Center of Montenegro, Podgorica, Montenegro

## Abstract

Montenegro has been at the top of the European antibiotic (AB) consumption list for a decade. Also, the invasive isolates of significant Gram “−” pathogens have one of the highest rates of resistance to key AB in Europe. A COVID-19 pandemic announced in 2020 had a significant impact on AB consumption globally. We analysed the consumption of AB in the pre-COVID (2019) and COVID-19 pandemic (2020, 2021, and 2022) period, and compared it with data on the resistance of Gram “−” invasive isolates of important pathogens to key AB. Data on total AB consumption in Montenegro (2011–2022) show that the growth rate in 2021 compared to 2020 was 14.04%, which is a statistically significantly higher value compared to previous years (*P* < .005, *Z*-value = 7.43). Additionally, there was a change in the structure of AB consumed, including hospital AB. Resistance of *Escherichia coli* to the third generation of cephalosporins increased significantly from 38% (9/24) in 2019 to 67% (16/24) in the COVID (2022) year (*χ*^2^ = 4.0904, *P* < .05). The highest rate of *Klebsiella pneumoniae* resistance to carbapenems was recorded in 2022, 47% (18/38), and was significantly higher compared to 2019 (17% (4/23)) (*χ*^2^ = 5.5838, *P* < .05). The rate of resistance to macrolides of *Staphylococcus aureus* strains increased significantly from 11% (101/920) in 2019 to 18% (134/735) in 2022 (*χ*^2^ = 17 640; *P* < .001). COVID-19 pandemic altered the resistance map of important pathogens to key antibiotics in Montenegro. A complete national stewardship program must be developed, and the surveillance should be rigorously enhanced and maintained.

## Introduction

In March 2020, the World Health Organization (WHO) declared a global COVID-19 pandemic, which claimed many lives around the globe over the next few years. Parallel to the COVID-19 pandemic, another pandemic was taking place, the AMR (antimicrobial resistance) pandemic, less noticeable but no less serious. It began long before the COVID-19 pandemic, continued after it, and relentlessly claims victims, so it is often called “silent tsunami”.

It has been unequivocally proven that the main driver of the development and spread of antibiotic resistance is the inappropriate, and overuse of antibiotics, especially in the treatment of infections, including viral infections. COVID-19 pandemic has raised additional concerns about the overuse of antibiotics and the consequences for AMR [1].

Although data showed less than 10% of hospitalized and outpatients with COVID-19 worldwide were diagnosed with a secondary bacterial infection requiring antibiotic therapy, it is estimated that 75% of patients received a prescription antibiotic [2–4]. Despite this, ESAC-net data show that outpatient antibiotic consumption in the EU/EAA declined during the pandemic, although the CDC reported the AMR in the US increased by 15% during 2019–2020 [5, 6]. Unlike in the rest of the world, the situation is different in the low-income countries. Additionally, antibiotics were used prophylactically during the COVID-19 surge, so there was a significant increase in their consumption [7–9].

It is important to assess the impact of the COVID-19 pandemic on antibiotic use and resistance at the national level in order to draw conclusions from the data obtained that could serve not only to guide policies when it comes to antibiotic consumption, but also to develop strategies for new scenarios that could be challenging in terms of antibiotic use and resistance control.

According to official data, Montenegro has been at the very top of European countries regarding antibiotic consumption for more than a decade, and according to WHO reports, the rate of resistance of invasive isolates of important Gram “−” pathogens to key antibiotics is among the highest in Europe [10, 11]. We examined the effect of COVID-19 on antibiotic consumption given the preexisting overuse of antibiotics and the effects on the resistance of the most common Gram “−” invasive isolates of important pathogens (*Klebsiella pneumoniae*, *Escherichia coli*, and *Acinetobacter* spp.), as well as pathogenic Gram “+” bacteria (*Staphylococcus aureus, Streptococcus pyogenes*) to key antibiotics.

## Methods

We analysed the consumption of antibiotics in the pre-COVID pandemic (2019) and COVID-19 (2020, 2021, and 2022) period, over the years and compared it with data on the resistance of Gram “−” invasive isolates of important pathogens (*K. pneumoniae*, *E. coli*, and *Acinetobacter* spp.) and Gram “+” bacteria (*S. aureus*, *S. pyogenes*) to key antibiotics, more consumed during the COVID pandemic.

Data on the total consumption of antibiotics in Montenegro expressed in daily defined doses per 1000 inhabitants per day (DDD/1000/day), for the period 2011–2022, were extracted from the Montenegrin Institute for Medicines and Medical Devices (CinMed), from where data on total and hospital consumption of the 10 most commonly used antibiotics for the period 2019–2022 were obtained.

Data on the resistance of the most common Gram “–” invasive isolates of important pathogens (*K. pneumoniae*, *E. coli*, and *Acinetobacter* spp.) were obtained from the Reference Laboratory for Monitoring Bacterial Resistance to Antibiotics of the Institute of Public Health of Montenegro (RL) for the period 2019–2023. The RL collects data on resistance according to the CAESAR methodology and sends them annually to the Dutch National Institute for Public Health and the Environment, which, based on the data obtained, prepares and reports to the RL on national resistance rates.

Data on the resistance of Gram “+” bacteria (*S. aureus*, *S. pyogenes*) to macrolides were collected from the laboratory information system of the Institute of Public Health in pre-covid 2019 and post-covid years: for *S. aureus* in 2022, and for *S. pyogenes* in 2022 and 2023. In 2019, data on sensitivity to macrolides were collected for 920, and in 2022 for 735 isolates of *S. aureus*. For *S. pyogenes*, macrolide susceptibility data were collected for 304 isolates in 2019, 350 isolates in 2022, and 422 isolates in 2023. Isolates for both bacterial species were isolated from different clinical non-replicated samples.

*Z*-test and *χ*^2^ test were used to statistically analyse the difference in antibiotic consumption and resistance.

## Results

**Figure 1.**
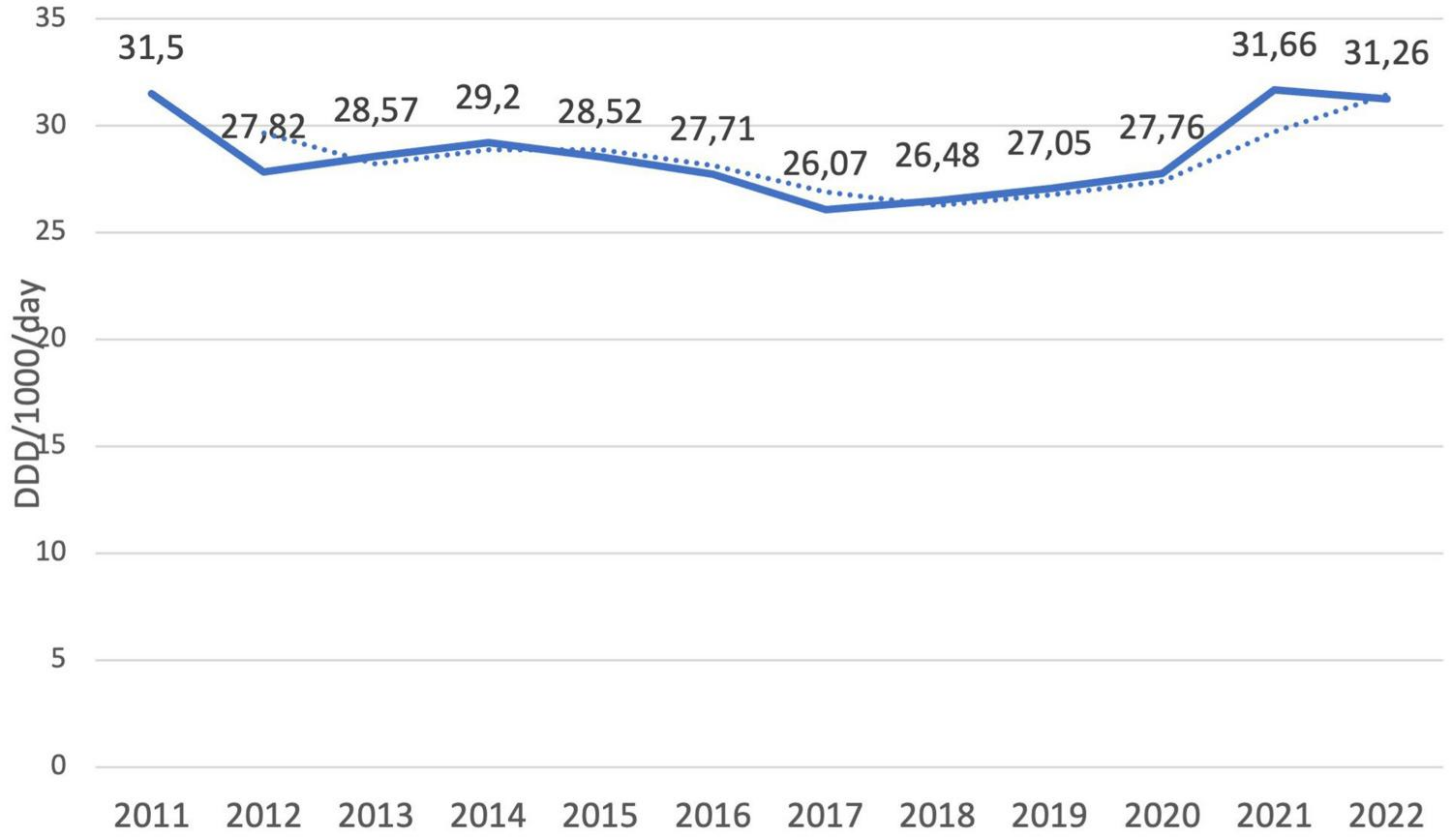
Total consumption of antibiotics in Montenegro expressed in DDD/1000/day.

Data on total antibiotic consumption in Montenegro from 2011 to 2022 show that the highest consumption was recorded in 2021 (31.66 DDD/1000/day), followed by 2011 (31.50 DDD/1000/day) (Fig. 1).

The lowest consumption was registered in 2017 and estimated to 26.07 DDD/1000/day.

The growth rate in 2021 compared to 2020 was 14.04%, which is significantly higher compared to the average rates of change in previous years, with a significance level of *P* < .005, *Z*-value = 7.43. The rates of change ranged from −11.67% (2011 compared to 2012) to 2.7% (2012 compared to 2013).

In addition to the increase in antibiotic consumption during COVID-19, there was also a change in the structure of antibiotics consumed (Table 1).

**Table 1.** Top 10 antibiotics in total consumption during 2019–2022 period

Rang	2019	DDD/1000/day	2020	DDD/1000/day	2021	DDD/1000/day	2022	DDD/1000/day
1	Amoxicillin	6.28	Azithromycin	6.19	Amoxicillin	4.94	Amoxicillin	5.83
2	Co-amoxiclav	2.72	Amoxicillin	4.86	Azithromycin	4.84	Azithromycin	5.11
3	Azithromycin	2.59	Cefixime	2.71	Cefixime	4.17	Cefixime	4.24
4	Cefixime	2.13	Ciprofloxacin	2.21	Ciprofloxacin	2.92	Co-amoxiclav	3.46
5	Cephalexin	2.02	Co-amoxiclav	1.81	Ceftriaxone	2.57	Ciprofloxacin	2.51
6	Ciprofloxacin	1.74	Cephalexin	1.67	Doxycycline	2.24	Cephalexin	1.85
7	Doxycycline	1.44	Doxycycline	1.56	Co-amoxiclav	2.2	Doxycycline	1.74
8	Erythromycin	1.27	Ceftriaxone	1.25	Cephalexin	1.38	Ceftriaxone	1.39
9	TMP/SMX	1.04	TMP/SMX	1.03	Clarithromycin	1.3	Clarithromycin	1.07
10	Ceftriaxone	1.03	Clarithromycin	0.93	TMP/SMX	0.94	TMP/SMX	0.92

Azithromycin consumption more than doubled in 2020 compared to the pre-COVID year, 2019 (6.19 DDD/1000/day vs. 2.59 DDD/1000/day), and remained at a high level in 2021 and 2022 (4.84 DDD/1000/day, 5.11 DDD/1000/day, respectively). The growth rate of azithromycin consumption in 2020 compared to 2019 has been shown to be the highest in 2020 (*P* < .001, *Z* = 10.74).

The data indicate that cefixim consumption was almost twice as high in 2021 (4.17 DDD/1000/day) and 2022 (4.24 DDD/1000/day) in comparison to pre-COVID consumption in 2019 (2.13 DDD/1000/day). The rate of cefixim consumption in 2021 and 2022 compared to 2019 significantly increased (*P* < .001, *Z* = 34.29).

In the observed four-year period, the consumption of ceftriaxone was the highest in the peak COVID year for Montenegro, 2021 (2.57 DDD/1000/day), 2.5 times higher than in 2019 (1.03 DDD/1000/day), which had statistical significance (*P* < .001, *Z* = 12.95).

The consumption of ciprofloxacin was also highest in 2021 (2.92 DDD/1000/day) and significantly higher compared to pre-COVID, 2019 when estimated to 1.74 DDD/1000/day (*P* < .001, *Z* = 4.02).

Although the largest part of antibiotic consumption belongs to outpatient consumption, some antibiotics are used only in hospitals, so we analysed hospital consumption (see Table 2). A structure of consumed antibiotics changed, comparing pre-COVID (2019) and COVID years (2020, 2021, and 2022).

**Table 2.** Top 10 antibiotics in hospital consumption in 2019–2022

Rang	2019	DDD/1000/day	2020	DDD/1000/day	2021	DDD/1000/day	2022	DDD/1000/day
1	Ceftriaxone	0.65	Ceftriaxone	0.61	Ceftriaxone	0.85	Ceftriaxone	0.59
2	Ciprofloxacin	0.13	Azithromycin	0.18	Meropenem	0.21	Metronidazole	0.13
3	Metronidazole	0.13	Ciprofloxacin	0.15	Ciprofloxacin	0.17	Ciprofloxacin	0.12
4	Azithromycin	0.09	Meropenem	0.12	Metronidazole	0.15	Azithromycin	0.09
5	Amikacin	0.07	Metronidazole	0.12	Azithromycin	0.14	Meropenem	0.09
6	Cefixime	0.06	Cefixime	0.07	Amikacin	0.10	Cefixime	0.08
7	Amoxicillin	0.05	Amikacin	0.07	Vancomycin	0.10	Amikacin	0.07
8	Meropenem	0.05	Vancomycin	0.06	Cefixime	0.08	Vancomycin	0.05
9	Co-amoxiclav	0.04	Doxycycline	0.04	Doxycycline	0.05	Amoxicillin	0.04
10	Vancomycin	0.04	Amoxicillin	0.04	Ceftazidime	0.03	Co-amoxiclav	0.04

In hospitals, the consumption of amoxicillin/beta lactam inhibitors decreased in 2020 and 2021, so this antibiotic was not among the 10 most commonly used antibiotics at that point, and appeared in 2022 in 10th place, when its consumption was the same as in pre-COVID, 2019 (0.04 DDD/1000/day).

Amoxicillin was also pushed out of the top 10 most commonly used antibiotics in 2021, and consumption in the other three years (2019, 2022, 2023) was approximately uniform (0.05 DDD/1000/day and 0.04 DDD/1000/day, respectively).

In 2020, there was a more than two-fold increase in meropenem consumption compared to 2019 (0.12 DDD/1000/day, 0.05 DDD/1000/day, respectively), and a peak was observed in 2021 (0.21 DDD/1000/day). It was shown that the growth rate of meropenem consumption in 2021 was significantly higher compared to 2019 (*P* < .01, *Z* = 2.83).

The consumption of ceftriaxone was the highest in the peak COVID year 2021 (0.85 DDD/1000/day) and significantly higher compared to the pre-COVID year, 2019, when it is estimated to 0.65 DDD/1000/day (*P* < .001, *Z* = 11.00).

The consumption of vancomycin in 2021 also increased significantly to 0.10 DDD/day/1000 compared to the pre-COVID year when it was 0.04 DDD/1000/day (*P* < .001, *Z* = 5.03), as well as ciprofloxacin (0.17 DDD/1000/day, 0.13 DDD/1000/day, respectively) (*P* < .001, *Z* = 3.02).

Although total consumption presents some kind of a reflection of outpatient consumption, some issues have emerged during the COVID-19 pandemic (Supplementary Material S1).

Although ceftriaxone is not intended for outpatient use in Montenegro (except as a continuation of hospital therapy), it appeared during the COVID-19 pandemic among the 10 most commonly prescribed antibiotics in outpatients. In 2020, it was in 10th place with 0.64 DDD/1000/day. In 2021, outpatient consumption nearly tripled (1.71 DDD/1000/day). With the pandemic subsiding in 2022, it was no longer among the 10 most commonly used antibiotics.

To see the effect of these changes in antibiotic consumption, we analysed data on the resistance of the most common invasive isolates of Gram “−” bacteria *E. coli*, *K. pneumoniae*, and *Acinetobacter* spp. to key antibiotics, including those more used during the COVID-19 pandemic (Table 3).

**Table 3.** Resistance rate of the most common Gram-negative invasive isolates to key antibiotics

		2019		2020		2021		2022		2023	
Bacterial species	Antimicrobial group/agent	*n*	%	*n*	%	*n*	%	*n*	%	*n*	%
*Escherichia coli*	Aminopenicillin (amoxicillin/ampicillin) resistance	23	74^f^	20	80^f^	7	NA	24	87^f^	35	77
	Third-generation cephalosporin (cefotaxime/ceftriaxone/ceftazidime) resistance	24	38^f^	20	40^f^	7	NA	24	67^f^	35	54
	Carbapenem (imipenem/meropenem) resistance	24	0^f^	20	0^f^	7	NA	24	0^f^	35	0
	Fluoroquinolone (ciprofloxacin/levofloxacin/ofloxacin) resistance	24	46^f^	20	40^f^	7	NA	24	54^f^	35	51
	Aminoglycoside (gentamicin/tobramycin) resistance	24	33^f^	20	30^f^	7	NA	24	42^f^	35	46
	Combined resistance to third-generation cephalosporins, fluoroquinolones and aminoglycosides	24	29^f^	20	15^f^	7	NA	24	25^f^	35	34
*Klebsiella pneumonia*	Third-generation cephalosporin (cefotaxime/ceftriaxone/ceftazidime) resistance	23	87^f^	29	86^f^	32	87	38	89	47	85
	Carbapenem (imipenem/meropenem) resistance	23	17^f^	29	14^f^	32	38	38	47	47	43
	Fluoroquinolone (ciprofloxacin/levofloxacin/ofloxacin) resistance	23	48^f^	29	62^f^	32	75	38	61	47	72
	Aminoglycoside (gentamicin/tobramycin) resistance	23	78^f^	29	86^f^	32	87	38	87	47	66
	Combined resistance to third-generation cephalosporins, fluoroquinolones and aminoglycosides	23	35^f^	29	62^f^	32	75	38	61	47	53
*Acinetobacter* spp.	Carbapenem (imipenem/meropenem) resistance	32	97	37	100	57	95	39	100	34	94
	Fluoroquinolone (ciprofloxacin/levofloxacin) resistance	32	97	37	100	57	96	39	97	34	100
	Aminoglycoside (gentamicin/tobramycin) resistance	32	81	37	92	57	89	39	95	34	100
	Combined resistance to carbapenems, fluoroquinolones and aminoglycosides	32	81	37	92	57	88	39	95	34	94

f = small denominator (<30 cases).

*Escherichia coli* resistance to the third generation of cephalosporins increased significantly from 38% (9/24) in 2019 to 67% (16/24) in 2022 (*χ*^2^ = 4.0904, *P* = .043127 < .05).

Invasive isolates of *K. pneumoniae* showed a high level of resistance to the third generation of cephalosporins (20/23; 87%) in pre-covid, 2019, and this level was maintained at approximate values throughout the observed period 2020, 2021, 2022, 2023 (86%, 87%, 89%, 85%, respectively).

The highest rate of *K. pneumoniae resistance* to carbapenems was recorded in 2022, 47% (18/38), and was significantly higher compared to 2019 when it was 17% (4/23) (*χ*^2^ = 5.5838, *P* = .018127 < 0.05).

The rate of resistance of *K. pneumoniae* to ciprofloxacin increased from 48% (11/23) in 2019 to 62% (18/29) in 2020 and did not decrease to or below 2019 value, during 2020–2023 period. A significant increase compared to the pre-COVID year was recorded in 2021 (75% (24/32)) and 2023 (72% (34/47)) (*χ*^2^ = 4.2702, *P* = .039; *χ*^2^ = 4.0421, *P* = .044, respectively).

A high resistance rate of *Acinetobacter* spp. to carbapenems (97%), fluoroquinolones (97%), and aminoglycosides (81%) was observed in the pre-COVID period and was maintained at a high level with minor variation in the observed period (2019–2023).

Since the COVID pandemic has shown the largest increase in the consumption of azithromycin, we examined the rates of resistance to macrolides of Gram “+” bacteria, *S. aureus* and *S. pyogenes*, for which this group of antibiotics is crucial.

Among 920 strains *of S. aureus* strains tested in 2019, 101 were resistant to macrolides, and of the 735 strains tested in 2022, 134 were resistant to macrolides. Data analysis showed that the resistance rate o macrolides of *S. aureus* strains increased significantly from 11% (101/920) in 2019 to 18% (134/735) in 2022 (*χ*^2^ = 17 640; *P* = .000027 < .001) (Supplementary Material S2).

The macrolide resistance rate of the tested *S. pyogenes* strains in 2019 and 2022 was unchanged at 4.3% (13/304; 15/350, respectively) (Supplementary Material S3).

In 2023, out of 422 strains tested, 33 (8%) were resistant to macrolides.

It is found that the difference in resistance to *S. pyogenes* macrolides in 2023 compared to 2019 and 2022 is at the borderline of statistical significance (*χ*^2^ = 3.7389; *P* = .05316).

## Discussion

Due to the difficulty to differentiate the ethiology of pneumonia (viral/bacterial), it was challenging worldwide to avoid antibiotics usage during COVID19 pandemic. Also, while searching for the best therapeutic option, knowing that secondary bacterial infection was not rare in patients with SARS-CoV2 infection, some guidelines recommended antibiotics as a treatment option [12]. Aware of the previously raised question of antibiotics availability in Montenegro as one of the reasons for the high antibiotics uptake, it is not surprising that our results pointed out the increased antibiotic consumption in Montenegro during the COVID19 pandemic [13].

Aligning with the global reports, the highest annual percentage change in antibiotics consumption rate was recorded in 2021 (14.04%), the second year of COVID [14].

Azitromycin was one of the first-line recommended medicines [15]. In the first year of COVID19 in Montenegro (2020), azithromycin was widely used, and the consumption was doubled in comparison to pre-COVID period. Similar sharp increase in azithromycin prescription was observed in nearby Croatia, where the consumption became triple [16]. It could be explained with the published hypothesis that due to its immunomodulatory effect, azithromycin has positive antiviral effect and it is associated with better COVID19 outcomes [17]. Afterward, systematic reviews reported against the association between azithromycin and clinical benefit in COVID-19 treatment [18]. In developed countries, as in Qatar, after the introduction of evidence based guidelines the azithromycin usage abated, which was not the case in Montenegro [19]. Hence, it is already assumed that Montenegrins have low knowledge on appropriate antibiotics use, which can affect antibiotics consumption, and reinforced with the patients anxiety and panic during pandemic, as well as pressure physicians experienced, could not result with a prudent antibiotics use [13].

Moreover, the structure of the other, empirically prescribed antibiotics changed. Amoxicillin consumption in hospitalized patients became subsided and the consumption of ceftriaxone and cefixime increased. Likewise, the consumption of ciprofloxacin, meropenem, and vancomycin increased during the COVID pandemic. Last listed (meropenem and vancomycin) are reserve antibiotics in Montenegro. This is an emerging finding, as those antimicrobials are broad-spectrum antibiotics which must not be used routinely.

The consumption of broad-spectrum antibiotics catalyses AMR, and therefore the literature inclines that COVID19 pandemic appears to accelerate the potential AMR pandemic [20]. CDC reported in 2022 an alarming increase in the following resistances: carbapenem-resistant *Acinetobacter*, carbapenem-resistant *Enterobacterales*, ESBL-producing *Enterobacterales*, vancomycin-resistant *Enterococcus*, multidrug-resistant *P. aeruginosa*, and methicillin-resistant *S. aureus* [21].

In Montenegro, in the pre-COVID period there was a high resistance rate of *Acinetobacter* to broad-spectrum antimicrobials such as carbapenems, fluorohinolones, and aminoglicozydes, and increased antibiotic consumption could not significantly change the already high resistance rate. The worrisome result is the resistance of *K. pneumoniae.* One year after increasing the consumption of broad-spectrum antibiotics, we registered a significant increase in *K. pneumoniae* resistance. *Klebsiella pneumoniae* resistance to third generation of cephalosporines was very high and persisted at the same level during COVID pandemic (about 87%), but resistance to carbapenems increased in 2022 (47% vs 17% in pre-COVID period) just one year after the peak of meropenem consumption. Also, *K. pneumoniae* resistance to fluoroquinolones increased significantly in 2021 (62% vs 48% in pre-COVID period), practically in the same year when a significant increase in the total consumption of ciprofloxacin, which is used in both outpatient and inpatient healthcare institutions, was observed.

As expected, the increased consumption of antibiotics had a slightly slower effect on gram-positive bacteria. Non-critical use of azithromycin, especially in 2020 when the peak was seen, brought in a significant increase of macrolide resistance of *S. aureus* and *S. pyogenes* in Montenegro. A significant increase in macrolide resistance was observed in *S. aureus* 2 years and *S. pyogenes* (to the level of the limit of statistical significance) 3 years after a significant increase in azithromycin consumption. Sánchez-Osuna *et al.* reported *S. aureus* adapted to COVID-19, due to the higher prevalence of methicillin (*mecA*) and macrolide (*msrA* and *mphC*) resistance genes [22].

This study has many limitations, starting with the small number of hemocultures, which our country is acclaimed for. That limits statistical analyses in population subgroups, such as groups based on age. It is important to highlight, AMR is a big threat in children, predominantly because of the irrational antibiotics prescribing and limited options of antibiotics due to the lack of trials in pediatric population. As a result, resistant bacteria spread quickly and cause 200 000 deaths in neonates annually, as WHO reported [23]. COVID19 outbreak exacerbated the problem in this vulnerable group [24].

In conclusion, COVID-19 pandemic altered the resistance map of important pathogens to key antibiotics in Montenegro, further encouraging the practice of inappropriate and overuse of antibiotics. This trend in resistance indicates an urgent need to develop a comprehensive national program for the rational use of antibiotics, in order to prevent further negative outcomes in potential new, similar to COVID19, challenges.

## Supplementary Material

ckaf167_Supplementary_Data

## Data Availability

All data are available upon request to the corresponding author. Key points COVID-19 pandemic encouraged further abuse of antibiotics in Montenegro. The resistance rate to newly developer and reserve antibiotics increased significantly. A comprehensive national stewardship program is needed.

## References

[1] Darwish Elhajji F , Abuhasheesh S, Al Rusasi A et al Overview of availability, cost, and affordability of antibiotics for adults in Jordan: an AWaRe classification perspective. Antibiotics (Basel) 2023; 12:1576. 10.3390/antibiotics12111576 37998778 PMC10668667

[2] Langford BJ , So M, Raybardhan S et al Antibiotic prescribing in patients with COVID-19: rapid review and meta-analysis. Clin Microbiol Infect 2021;27:520–31. 33418017 10.1016/j.cmi.2020.12.018 PMC7785281

[3] Rose AN , Baggs J, Wolford H, et al Trends in antibiotic use in United States hospitals during the coronavirus disease 2019 pandemic. Open Forum Infect Dis 2021;8:ofab236. 34226869 10.1093/ofid/ofab236 PMC8244661

[4] Russell CD , Fairfield CJ, Drake TM, et al; ISARIC4C investigators. Co-infections, secondary infections, and antimicrobial use in patients hospitalised with COVID-19 during the first pandemic wave from the ISARIC WHO CCP-UK study: a multicentre, prospective cohort study. Lancet Microbe 2021;2:e354–65. 34100002 10.1016/S2666-5247(21)00090-2 PMC8172149

[5] Högberg LD , Vlahović-Palčevski V, Pereira C et al, ESAC-Net Study Group. Decrease in community antibiotic consumption during the COVID-19 pandemic, EU/EEA, 2020. Eurosurveillance 2021;26:1–5. 10.2807/1560-7917.ES.2021.26.46.2101020 PMC8603403 34794534

[6] Centers for Disease Control and Prevention. COVID-19: US Impact on Antimicrobial Resistance, Special Report 2022. Atlanta, GA: US Department of Health and Human Services; 2022.

[7] Sulis G , Batomen B, Kotwani A, Pai M, Gandra S. Sales of antibiotics and hydroxychloroquine in India during the COVID-19 epidemic: an interrupted time series analysis. PLoS Med 2021;18:e1003682. 34197449 10.1371/journal.pmed.1003682 PMC8248656

[8] Quincho-Lopez A , Benites-Ibarra CA, Hilario-Gomez MM, Quijano-Escate R, Taype-Rondan A. Self-medication practices to prevent or manage COVID-19: a systematic review. PLoS One. 2021;16:e0259317. 34727126 10.1371/journal.pone.0259317 PMC8562851

[9] Adebisi YA , Jimoh ND, Ogunkola IO et al The use of antibiotics in COVID-19 management: a rapid review of national treatment guidelines in 10 African countries. Trop Med Health 2021;49:51. 34162445 10.1186/s41182-021-00344-w PMC8220112

[10] Versporten A , Bolokhovets G, Ghazaryan L et al; WHO/Europe-ESAC Project Group. Antibiotic use in Eastern Europe: a cross-national database study in coordination with the WHO Regional Office for Europe. Lancet Infect Dis 2014;14:381–7. 10.1016/S1473-3099(14)70071-4 24657114

[11] Antimicrobial Resistance Surveillance in Europe 2023—2021 Data. Stockholm: European Centre for Disease Prevention and Control and World Health Organization; 2023.

[12] Rehman S. A parallel and silent emerging pandemic: antimicrobial resistance (AMR) amid COVID-19 pandemic. J Infect Public Health 2023;16:611–7. 10.1016/j.jiph.2023.02.021 36857834 PMC9942450

[13] Raicevic M , Labovic Barjaktarovic S, Milic D et al Public knowledge, attitudes, and practices regarding antibiotics use and resistance in Montenegro. Eur J Public Health 2025;35:290–4. 10.1093/eurpub/ckae213 39833135 PMC11967873

[14] Klein EY , Impalli I, Poleon S et al Global trends in antibiotic consumption during 2016–2023 and future projections through 2030. Proc Natl Acad Sci U S A 2024; 121:e2411919121. 10.1073/pnas.2411919121 39556760 PMC11626136

[15] Schwartz RA , Suskind RM. Azithromycin and COVID-19: prompt early use at first signs of this infection in adults and children, an approach worthy of consideration. Dermatol Ther 2020;33:e13785. 10.1111/dth.13785 32510734 PMC7300563

[16] Bogdanić N , Močibob L, Vidović T et al Correction: azithromycin consumption during the COVID-19 pandemic in Croatia, 2020. PLoS One 2022;17:e0272324. 10.1371/journal.pone.0272324 35881620 PMC9321362

[17] Gautret P , Lagier JC, Parola P et al RETRACTED: hydroxychloroquine and azithromycin as a treatment of COVID-19: results of an open-label non-randomized clinical trial. Int J Antimicrob Agents 2020;56:105949. 10.1016/j.ijantimicag.2020.105949 32205204 PMC7102549

[18] Ayerbe L , Risco-Risco C, Forgnone I et al Azithromycin in patients with COVID-19: a systematic review and meta-analysis. J Antimicrob Chemother 2022;77:303–9. 10.1093/jac/dkab404 34791330 PMC8690025

[19] Butt AA , Shams S, Nafady-Hego H et al Azithromycin use before and during the COVID-19 pandemic and the impact of implementing national evidence-based guidelines in Qatar. Clin Epidemiol Glob Health 2024;30:101843.

[20] Ansari S , Hays JP, Kemp A et al; Global AMR Insights Ambassador Network. The potential impact of the COVID-19 pandemic on global antimicrobial and biocide resistance: an AMR insights global perspective. JAC Antimicrob Resist 2021;3:dlab038. 10.1093/jacamr/dlab038 34192258 PMC8083476

[21] US Centers for Disease Control and Prevention. COVID-19: U.S. Impact on antimicrobial resistance, special report 2022 [Internet]. National Center for Emerging and Zoonotic Infectious Diseases; 2022. https://stacks.cdc.gov/view/cdc/117915.

[22] Sánchez-Osuna M , Pedrosa M, Bierge P et al Genomic analysis of *Staphylococcus aureus* isolates from bacteremia reveals genetic features associated with the COVID-19 pandemic. iScience 2024;27:110402. 39108736 10.1016/j.isci.2024.110402 PMC11301081

[23] Romandini A , Pani A, Schenardi PA et al Antibiotic resistance in pediatric infections: global emerging threats, prediciting the near future. Antibiotics (Basel) 2021;10:393. 33917430 10.3390/antibiotics10040393 PMC8067449

[24] Karun A , Kaur RJ, Charan J et al Impact of COVID-19 on antimicrobial resistance in pediatric population: a narrative review. Curr Pharmacol Rep 2022;8:365–75. 35789932 10.1007/s40495-022-00298-5 PMC9244284

