# Supplementary material for: The impact of the COVID-19 pandemic on the antibiotic consumption and resistance in Montenegro
Source: Eur J Public Health. 2025 Sep 13;35(6):1295–9. doi: 10.1093/eurpub/ckaf167 (PMC12707471; doi:10.1093/eurpub/ckaf167)
Supplement: ckaf167_Supplementary_Data [file ckaf167_supplementary_data.zip › ejph-2025-05-om-0377-File003.docx]

**Table S1.** Top 10 antibiotics in outpatient consumption in 2019-2022

| Rang | 2019 | DDD/  1000/  day | 2020 | DDD/  1000/  day | 2021 | DDD/  1000/  day | 2022 | DDD/  1000/  day |
| --- | --- | --- | --- | --- | --- | --- | --- | --- |
| 1 | amoxicillin | 6.23 | azithromycin | 6.02 | amoxicillin | 4.91 | amoxicillin | 5.79 |
| 2 | co-amoxiclav | 2.67 | amoxicillin | 4.82 | azithromycin | 4.71 | azithromycin | 5.02 |
| 3 | azithromycin | 2.50 | cefixime | 2.64 | cefixime | 4.08 | cefixime | 4.16 |
| 4 | cefixime | 2.07 | ciprofloxacin | 2.06 | ciprofloxacin | 2.75 | co-amoxiclav | 3.42 |
| 5 | cefalexin | 2.01 | co-amoxiclav | 1.78 | doxycycline | 2.19 | ciprofloxacin | 2.39 |
| 6 | ciprofloxacin | 1.61 | cefalexin | 1.67 | co-amoxiclav | 2.16 | cefalexin | 1.84 |
| 7 | doxycycline | 1.41 | doxycycline | 1.52 | ceftriaxone | 1.71 | doxycycline | 1.71 |
| 8 | erythromycin | 1.26 | TMP/SMX | 1.01 | cefalexin | 1.37 | clarithromycin | 1.05 |
| 9 | TMP/SMX | 1.02 | clarithromycin | 0.90 | clarithromycin | 1.28 | TMP/SMX | 0.90 |
| 10 | clarithromycin | 0.98 | ceftriaxone | 0.64 | TMP/SMX | 0.92 | erithromycin | 0.85 |
