# Supplementary material for: The impact of the COVID-19 pandemic on the antibiotic consumption and resistance in Montenegro
Source: Eur J Public Health. 2025 Sep 13;35(6):1295–9. doi: 10.1093/eurpub/ckaf167 (PMC12707471; doi:10.1093/eurpub/ckaf167)
Supplement: ckaf167_Supplementary_Data [file ckaf167_supplementary_data.zip › ejph-2025-05-om-0377-File004.docx]

**Figure S2.** Resistance of *Staphylococcus aureus* to macrolides in 2019 and 2022
